# Novelty Enhances Visual Perception

**DOI:** 10.1371/journal.pone.0050599

**Published:** 2012-12-05

**Authors:** Judith Schomaker, Martijn Meeter

**Affiliations:** Department of Cognitive Psychology, VU University, Weesp, Noord-Holland, The Netherlands; Radboud University Nijmegen, Netherlands

## Abstract

The effects of novelty on low-level visual perception were investigated in two experiments using a two-alternative forced-choice tilt detection task. A target, consisting of a Gabor patch, was preceded by a cue that was either a novel or a familiar fractal image. Participants had to indicate whether the Gabor stimulus was vertically oriented or slightly tilted. In the first experiment tilt angle was manipulated; in the second contrast of the Gabor patch was varied. In the first, we found that sensitivity was enhanced after a novel compared to a familiar cue, and in the second we found sensitivity to be enhanced for novel cues in later experimental blocks when participants became more and more familiarized with the familiar cue. These effects were not caused by a shift in the response criterion. This shows for the first time that novel stimuli affect low-level characteristics of perception. We suggest that novelty can elicit a transient attentional response, thereby enhancing perception.

## Introduction

Detection of new information is crucial for adapting behavior to the present circumstances - therefore the human brain is tuned towards novelty [1]. Novel stimuli often receive priority over other stimuli by reflexively attracting attention [2], [3], evoking the so-called orienting reflex or orienting response [1], [4], [5], [6]. The allocation of attentional resources to novel information is also reflected by the electrophysiological response commonly found to novel stimuli; the novelty P3 event-related potential component [7], [8]. Auditory novelty has been shown to have an effect on early event-related potential components: Strikingly, deviance can be detected as early as 30–40 ms post-stimulus [9], [10], [11]. Despite, or maybe because of, these automatic attentional mechanisms novelty is known to affect several cognitive processes. For example, words presented in deviant (novel) fonts are better remembered than words presented in standard fonts [12]. Novelty can also boost motivation to explore the environment, resulting in the so-called exploration bonus [13]. However, so far no benefit has been found of novelty on perception, which would be expected if novelty has a role in preparing us for changing circumstances.

There is another reason to expect novelty to affect perceptual processing. Emotional stimuli are known to enhance early perceptual processing (for a review see [14]). In cueing paradigms emotional stimuli have been reported to enhance early visual perception [15], [16], [17]. For example, Bocanegra and Zeelenberg (2009) found that the presentation of a fearful face improves sensitivity for low-spatial-frequency stimuli: immediately after presentation of such a face, participants can detect such stimuli at higher rates than after presentation of a neutral face. Bocanegra and Zeelenberg (2009), and others using similar paradigms, explained this by assuming that emotional significance enhances the attentional response generated by the cue, benefiting subsequent stimuli. The orienting response to novel stimuli has been linked to the activation of fundamental motivational circuits also associated with the attentional processes related to emotional stimuli [18]. Furthermore, novel stimuli are known to activate brain regions also related to emotional processing, specifically the amygdala [19], [20], [21], [22], a nucleus in the medial temporal lobe believed to have a crucial role in the evaluation of emotional significance [23]. Possibly, the enhancing effects that emotional stimuli have on perception are also present for novel stimuli activating the same emotional system.

To investigate this hypothesis we investigated novelty’s effects on visual perception in a cueing paradigm. We did this by manipulating the novelty versus familiarity of cue stimuli that were all fractals. Every participant was familiarized with one of the fractals by viewing this stimulus for at least 20 seconds. In the subsequent blocks, 50% of cues consisted of this one familiar stimulus, familiarizing participants even more with it. The other category of stimuli, the novels, were all presented only once during the experiment to guarantee their novelty. The novel/familiar cues were presented centrally. The effects of the cues were measured in a tilt detection task. Performance on such a task is facilitated by contrast [24], [25], [26], as mediated by transient attention. Transient attention is an automatic and stimulus-driven process which is believed to enhance the visibility of stimuli by strengthening signal strength, and thereby increasing performance on a range of visual perceptual tasks [27], [28], [29], [30], [31], [32], [33]. As emotion in the emotional cueing paradigms described above, we expected that perceptual contrast would be enhanced by novelty via such an attentional mechanism [15], [16].

In the first experiment we investigated the effects of novel cues on perception for tilts of different angles. In experiment 2, we elaborated on this design by measuring contrast sensitivity. In both experiments we predicted that novel cues would enhance sensitivity of perception of the target compared to familiar cues.

## Methods

### Experiment 1

#### Participants

17 participants (12 female; 15 right-handed) participated in the experiment on a voluntary basis. All participants were naïve to the aim of the study and signed written informed consent before participation. The experiments were performed in accordance to the ethical standards laid down in the 1964 Declaration of Helsinki and approved by the faculty ethical committee, the Scientific and Ethical Review Board of the Faculty of Psychology and Education (VCWE). Participants were paid 6–7 Euros of compensation or were given course credits. The participants all had normal or corrected-to-normal vision.

#### Stimuli and apparatus

The stimuli were presented on a 19 inch CRT monitor (1024x768 pixels) at a viewing distance of about 75 cm using E-Prime programming software (Psychology Software Tools Inc., Pittsburgh, PA, USA). The refresh rate of the screen was 120 Hz.

All stimuli were presented in the center of the screen, on top of a silver background (luminance CIE(.34,.39), 61.60 cd/m^2^). A trial started with a black fixation cross, followed by a cue display consisting of a centrally presented fractal image (10.2°×10.2°). Novel/familiar fractal stimuli were generated by iterative mathematical computations using the open-source program ChaosPro 4.0 (http://chaospro.de). These fractals do not represent anything and are guaranteed to be new to the participants (similar to the fractal images used by [34]). The target display consisted of a 9.5°×9.5° Gabor patch, a sinusoidal grating with a Gaussian envelope with a low spatial frequency; 0.6 cycles/degree. The colors of the grating were black (luminance CIE(.00,.00), 0 cd/m^2^) and white (luminance CIE(.31,.34), 89.31 cd/m^2^). These were generated using an online Gabor patch generator created by Sebastiaan Mathot (http://www.cogsci.nl/software/online-gabor-patch-generator). Gabor patches either were tilted (clockwise or counterclockwise) or vertical. Tilts could be of 1°, 2°, 3°, 4°, each occurring on 96 trials. The untilted target (0°) occurred as often as the four tilts together, that is, on 384 trials. The tilt of the target Gabor was randomized over trials.

#### Procedure

For every participant one fractal was randomly chosen to become familiarized. This ‘familiar’ image was presented for 20 seconds prior to the experiment, in order for participants to become familiarized with it. The ‘novel’ images were all unique fractals unknown to the observer.

Before the experiments participants were told that accuracy on the task was most important, and that speeded responses were not required. In addition participants were instructed that a fixation cross would be presented between trials and stimuli, and that they should always fixate their eyes on this cross. All participants performed 48 training trials. Then 6 experimental blocks were completed of 128 trials, with a short break in the middle and at the end of each block. Half the trials had familiar cues, and half had novel cues. All 816 trials were performed in about 40 minutes.

**Figure 1:**
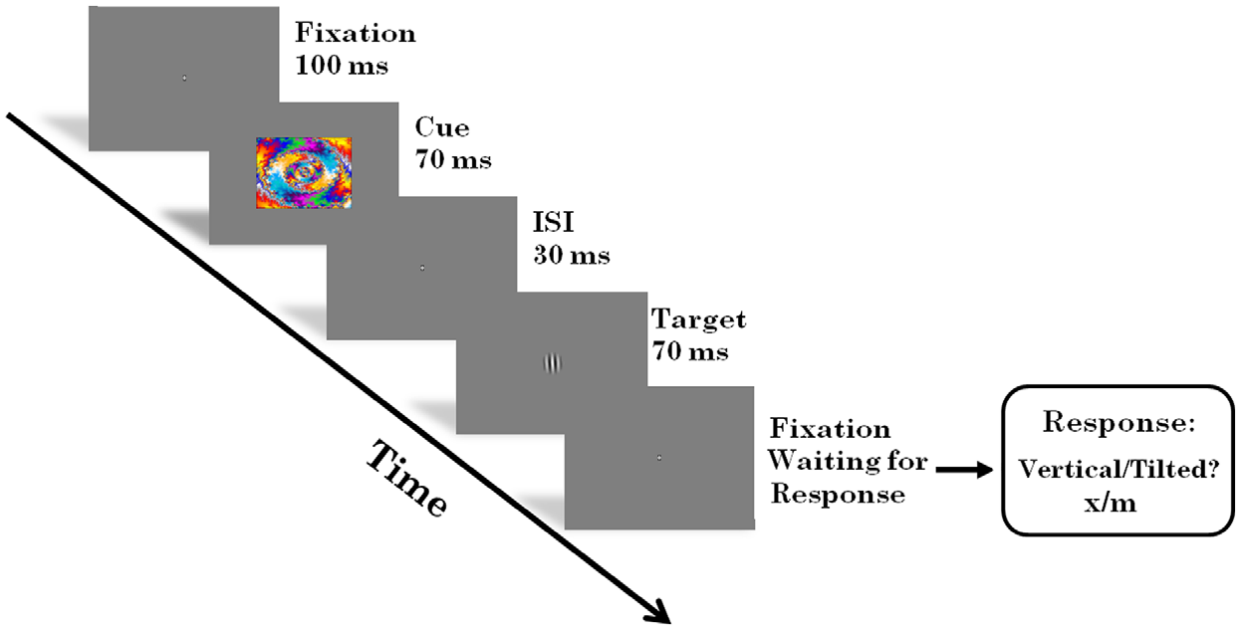
Example experimental trial. In Experiment 1 the target could be tilted either 0, 1, 2, 3, or 4 degrees. In Experiment 2 the target could be tilted with any of these angles, determined by the participant’s performance in the practice trials. In addition the contrast of Experiment 2 targets was varied in nine log increments.

Every trial started with the onset of a fixation cross presented for 1000 ms. After fixation a cue, either a novel or familiar fractal image, was briefly presented for 70 ms. Subsequently, the fixation cross was presented again for 30 ms. Then the target, consisting of a centrally presented Gabor patch (vertical/tilted), was presented for 70 ms. The timing of the stimuli was taken from a cueing paradigm used to investigate the effects of emotion on early vision [16]. Following the target the fixation cross was presented, until a response was given by the participant. See Figure 1 for an example stimulus sequence.

We used a two-alternative forced-choice (2AFC) task in which participants pressed “x” to indicate that the target Gabor was oriented vertically, or “m” to indicate that it was tilted (either left or right). Visual feedback was given to correct (“Correct”) and incorrect (“Incorrect”) trials for 1000 ms after a response was given.

*d’* was calculated for every condition for every participant: *d’* = *Z*(hit rate) − *Z*(false alarm rate). Hit rate was defined as the proportion of correctly identified tilted targets, whereas false alarm rate was defined as the proportion of untilted targets falsely identified as tilted targets. The effects of cue type (novel or familiar) on perception, as measured by the *d’* of tilt detection, was investigated by conducting a repeated measures ANOVA with Cue Type and Orientation of the target Gabor patch as factors. A second repeated measures ANOVA was conducted with Cue Type and Block (experimental block) to investigate how the effects developed during the experiment. Interaction effects were investigated in more detail using post-hoc paired-sample *t*-tests. The effects of cue type in a previous trial on accuracy were investigated by performing paired *t*-tests comparing trials on which a familiar cue was either preceded by a familiar or a novel cue on the previous trial.

In addition, response bias was calculated for every condition and participant: β = -(Z(hit rate) – Z(false alarm rate))/2. Since there were no separate lure conditions for each angle, bias was only computed for the two cue type conditions and for different blocks. The same ANOVA as for *d’* were performed for response bias across condition and block. If necessary Greenhouse Geisser correction was applied.

#### Results

**Figure 2:**
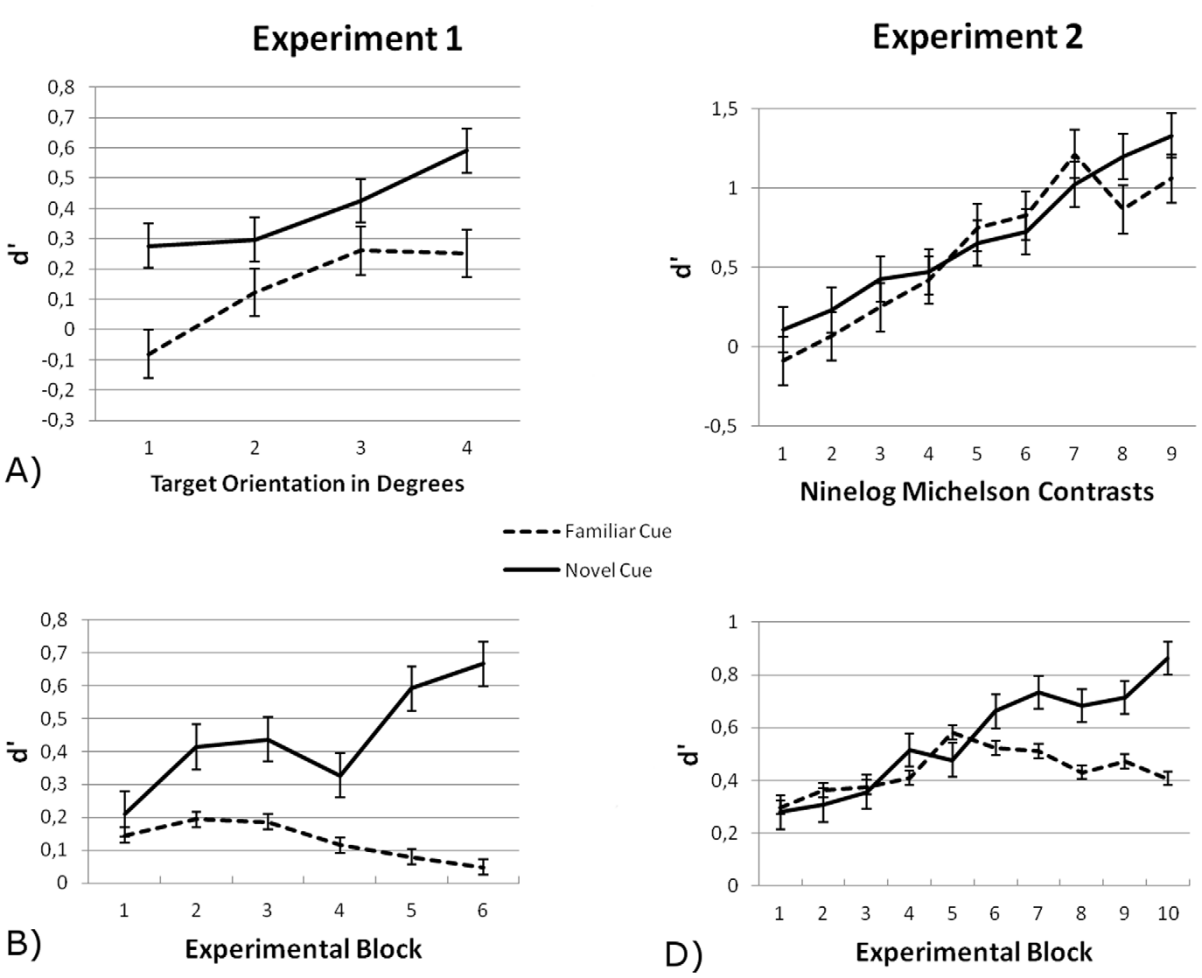
Accuracy of tilt detection measured by *d’* for novel and familiar cues as a function of A) target orientation in Experiment 1, B) experimental blocks in Experiment 1, C) contrast in Experiment 2, and D) experimental blocks in Experiment 2. Error bars reflect standard errors of the mean (between-subject).

For every participant and condition hit rates and false alarm rates that exceeded.975 were clipped to.975 to prevent creating extreme outliers and allowing *d’* to be calculated Figure 2A shows the average accuracy (as measured by *d’*) of tilt detection as a function of the target orientation in degrees. Participants perceived targets better with greater deviance from vertical alignment – main effect of tilt, *F*(3,48) = 4.06, *p = *.012, η^2^ = .20. More importantly, there was a main effect of cue type, *F*(1,16) = 7.24, *p = *.016, η^2^ = .31: As hypothesized, targets presented after novel cues were better perceived than targets presented after familiar cues. There was a trend towards an interaction between cue type and tilt, *F*(1, 48) = 2.55, *p = *.067, η^2^ = .14.

In the ANOVA with cue type and experimental block as within-subject variables, there was again a main effect of cue type on *d’*, with better performance for targets cued by novels compared by familiars, *F*(1,16) = 7.85, *p = *.013, η^2^ = .33. Block did not affect *d’* (*F* <1, tested with linear contrasts). There was a trend towards a larger cue effect on later blocks, *F*(1,16) = 3.34, *p = *.086, η^2^ = .17, tested with linear contrasts, suggesting that the benefit for novel cues was larger later in the experiment than it was in the early blocks of the experiment. Figure 2B shows how the effects of cue type develop over experimental blocks.

Familiar cues are often preceded by trials on which the same cue was shown. To investigate whether a possibly subsequent low-level adaptation to the familiar cue could have caused a reduction in the attentional resources allocated to the target, trials on which a familiar was preceded by either another familiar cue (mean *d’* = .08) or by a novel cue (mean *d’* = .17) were compared, but preceding cue type did not affect accuracy, *t*(16) = .96, *p = *.350.

**Figure 3:**
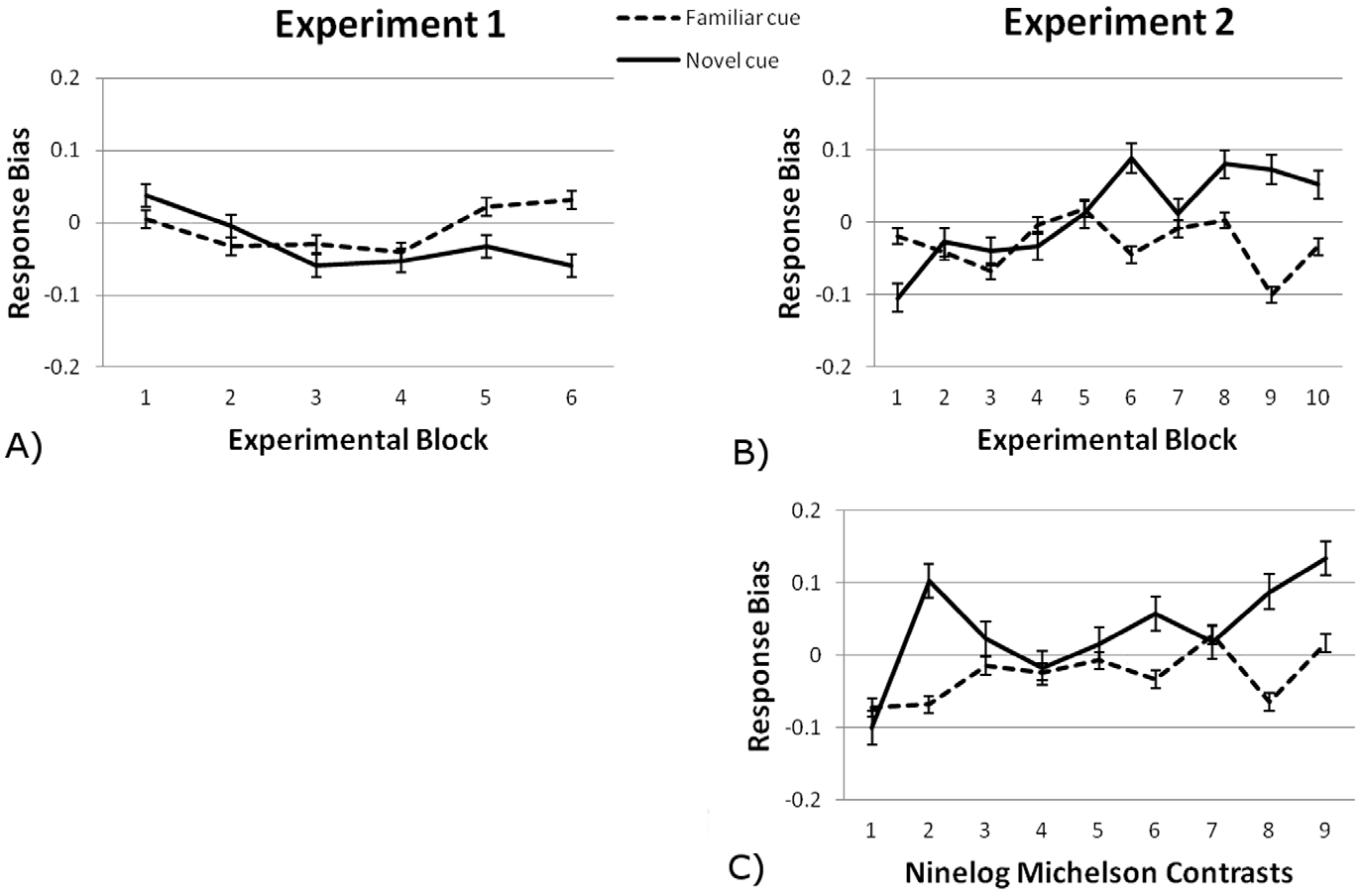
Response bias for novel and familiar cues as a function of A) experimental block in Experiment 1, B) experimental block in Experiment 2, C) contrast in Experiment 2. Error bars reflect standard errors of the mean (between-subject).

There was no main effect of cue type on response bias, *F*(1,16) = .05, *p = *.822, η^2^ = .003, neither of Block, *F*(5,80) = .91, *p = *.48, η^2^ = .05. Furthermore, cue type and block did not interact, *F*(5,50) = 1.09, *p = *.37, η^2^ = .06. So, the experimental factors cue type and experimental block did not alter the response criteria of the participants. See Figure 3 for response bias means for novel and familiar cues for each experimental block.

#### Discussion

As predicted novel cues enhanced tilt detection compared to familiar cues. More specifically, orientation detection was improved when the target was preceded by a novel cue. This effect seemed to become stronger during the experiment, although statistical evidence for this remained at the level of a trend. The effect cannot be caused by forward masking of the target by the cue stimulus, since novel and familiar stimuli did not differ in stimulus properties. Familiar cues were often preceded by trials in which the same cue was used, while this was by definition never the case for the unique novel cues. Although it could theoretically lead to adaptation to familiar cues, repetition of the familiar cue had no effect on performance. This suggests that it is the familiarity or novelty of the cue, and not some short-range effect, that causes the differences found in the experiment.

No differences in response criterion were found, suggesting that novelty improved performance on the tilt detection task by enhancing sensitivity.

### Experiment 2

To replicate and extend the findings of Experiment 1, we performed a new experiment using a similar cueing task, now varying the contrast of the target Gabor patches.

#### Participants

17 participants (15 female; 15 right-handed) participated in the experiment on a voluntary basis. All participants were naïve to the aim of the study and signed written informed consent before participation. Participants were paid 9 Euros of compensation or were given course credits. The participants all had normal or corrected-to-normal vision.

#### Stimuli and apparatus

The stimuli and apparatus were the same as in Experiment 1; however, now the participants were presented with Gabor patches from only one tilt angle (determined by practice block performance – see below). The task was identical to the one in Experiment 1, that is, participants had to indicate whether the briefly presented target Gabor patch was either tilted (button press “m”) or untilted (button press “x”). Accuracy, but not speed was emphasized. The new Gabor patches were created with a Matlab script, and had Michelson contrasts in nine log increments (from 2 to 20% contrast) compared to only one contrast in Experiment 1.

#### Procedure

The procedure was similar to the procedure in Experiment 1. However, the angle of the Gabor patches was now determined on the performance on the practice trials for every participant. The angle could either be 1, 2, 3, 4, 5, or 6 degrees (all presented 16 times). The smallest angle was chosen for which performance >.8. If performance did not reach.8 a 6 degree tilt was used in the remainder of the experiment. Participants performed 96 practice trials and a total of 1440 experimental trials. Half of the experimental trials contained tilted and the other half untilted targets (720 trials each). Participants could take a break after every 72 trials. The experiment lasted about 80 minutes.

#### Results and discussion

The effects of cue type on tilt detection (as measured by *d’*) was investigated by conducting a repeated measures ANOVA with Cue Type (novel/familiar) and Contrast (nine log increments of Michelson contrast) of the target Gabor patch as factors. A second repeated measures ANOVA was conducted with Cue Type and Block (experimental block) to investigate how the effects developed during the experiment. Figure 2C shows the average accuracy of tilt detection as a function of the nine log Michelson contrasts.

The contrast of the target exhibited a strong effect on performance, *F*(1.43,22.90) = 6.18, *p = *.013, η^2^ = .28. Participants better perceived the targets with higher contrast. There was no main effect of cue type, *F*(1,16) = .44, *p = *.52, η^2^ = .027, and cue type and contrast did not interact, *F*(8,128) = .90, *p = *.52, η^2^ = .05.

In our analysis with Block and Cue type as factors, there was again no main effect of cue type on tilt detection, *F*(1,16) = 1.01, *p = *.33, η^2^ = .06. Experimental block did affect performance, *F*(1,16) = 6.42, *p = *.022, η^2^ = .29, tested with linear contrasts, showing that performance increased over blocks. Moreover, there was an interaction between the linear block effect and cue type, *F*(1,16) = 7.72, *p = *.013, η^2^ = .33. To further investigate the interaction, separate ANOVAs were performed for the cue types. These ANOVAs showed that experimental block exhibited a strong linear effect for novel cues, *F*(1,16) = 16.67, *p = *.001, η^2^ = .51, but no effect for familiar cues *F*(1,16) = 1.44, *p = *.248, η^2^ = .08. See Figure 2C for accuracy as a function of experimental block. As in Experiment 1 contrast sensitivity increased as a function of experimental block: during the experiment participants’ visual perception became better, but only when the target was preceded by a novel rather than by a familiar cue. Whether a familiar cue was preceded by a trial with a novel (mean *d’* = .42) or familiar cue (mean *d’* = .43) did not have an effect on accuracy, *t*(16) = .13, *p = *.902, suggesting again that low-level adaptation to the familiar cues played no role in the results.

The cues did not alter participants’ response criterion, *F*(1,16) = .46, *p = *.51, η^2^<.03; neither did contrast, *F*(3.33,53.27) = 1.73, *p*<.097, η^2^ = .10. See Figure 3 for the mean response bias per condition. Cue type and contrast did show a linear interaction, *F*(1,16) = 5.95, *p = *.027, η^2^ = .27: for novel cues participants adopted a more conservative response strategy (fewer false alarms) with higher contrasts but not with lower contrasts, but performance was nonetheless increased.

Also in our analysis with blocks as factor there was no main effect of cue type on response bias, *F*(1,16) = .51, *p = *.485, η^2^ = .031. Neither was there one of Block, *F*(3.44,55.04) = .41, *p = *.77, η^2^ = .03, nor did cue type and block interact, *F*(9,144) = .89, *p* = .533, η^2^ = .05. So the experimental factors cue type and experimental block did not alter the response criteria of the participants.

## General Discussion

Novelty has long been known to have distracting effects by evoking an orienting response towards new things in the environment [2], [35], [36], [37]. However, more recently there have been indications that novel information can also have facilitating effects on target processing. An event-related potential (ERP) study showed that novel sounds can influence a late stage of processing, enhancing the event-related visual P3 component to visual targets [38], and lowering response times to these targets. Interestingly the sounds that exhibited this facilitatory effect evoked the same electrophysiological responses as typically evoked by distracting sounds (such as mismatch negativity and novelty P3). This finding suggests that the novelty-evoked P3 component does not only reflect attentional allocation and distraction but also encompasses an alerting response [38].

In the present study, the effect of novel stimuli on visual perception was investigated in two experiments. If novelty would enhance visual perception by recruiting attentional resources, one would expect to find higher sensitivity on the trials in which the cue was novel. The first experiment revealed better tilt detection after novel cues for different degrees of tilt. These effects tended to grow stronger over experimental blocks (statistical trend). Possibly the familiar cue became more familiar over repeated presentations, making the novels stand out more and increasing their effects on perception. We did not find a shift of the response criterion, suggesting that the sensitivity was enhanced for novel cues, rather than that participants changed their response behavior. In the second experiment the effect of novel compared to familiar cues on contrast sensitivity was investigated. Similarly, it was found that novel cues facilitated visual perception in later experimental blocks; that is, contrast sensitivity as measured by *d’* was enhanced on trials with novel cues later in the experiment. Furthermore, participants adopted a more conservative response criterion on trials with novel cues for targets with higher contrast, still showing enhanced performance on the tilt detection task. Such a shift towards a more conservative response criterion was not found for familiar cues.

We hypothesize that the enhancing effects of novelty are mediated by transient attention [39], [40], probably via a mechanism similar to that underlying enhancement of perception by emotional stimuli. Transient attention is known to enhance contrast sensitivity by modulating signal strength [25], [41], [42], although there is still some debate about this (e.g. see [43]). Moreover, covert transient attention can modulate contrast sensitivity in early visual areas in the brain on a variety of visual tasks [24], [25], [31], [44], [45], [46], [47], and spatial resolution [42], [48], [49].

As proposed by Weichselgartner and Sperling [40], target detection sets off an immediate attentional response that temporarily enhances perception at the triggering location. Much subsequent work, for example in the literature on the attentional blink (see [50]), has shown that it is not just a target that can set off such a response, but anything that is significant to the observer. The current work suggests that the appearance of a novel stimulus is such a significant event, setting off such an attentional response.

There is an alternative explanation of our findings. Possibly, repeated presentation of the familiar cues reduced attentional orienting due to low-level adaptation. To test this alternative, we reasoned that there should be some release from adaptation if a familiar follows a novel cue trial, as compared to familiar cue trial. In both experiments, we found no evidence for such an effect. However, it might still be that the better perception of the target after novel cues relative to familiar cues might not reflect an enhancement after a novel cue, but a normal attentional response to the novel cues compared to a reduced attentional response to the repeated familiars. This explanation would be the other side of the coin relative to the one given above: The significance of novelty to the brain might be the absence of habituation. It is difficult to disentangle these effects, since both interpretations would lead to almost the same pattern of results – better performance after novel compared to familiar cues. However, a reduced attentional orienting account would predict reduced performance for the familiar cues over experimental blocks when familiarity increased. In contrast, in both Experiment 1 and 2 such an effect of the familiar cue over experimental blocks was not significant, whereas the novelty effect was strengthened over experimental blocks in Experiment 2. These findings suggest that increased familiarity did not affect performance as strongly as novelty did.

One way in which cue novelty could affect performance on the task is by suppressing eye movements. If participants do not fixate at the center of the screen throughout the trial, their performance at the tilt detection task would be affected. Novelty could help participants fixate through increased visual stimulation. However, such an explanation would be hard to maintain given the timing in our experiments: the interval from the onset of the cue to the offset of the target stimulus was only 170 ms, shorter than all but the fastest express saccades.

Emotional stimuli already have already been shown to exhibit beneficiary effects on early visual perception, but this is the first time that novelty is shown to exhibit such effects. It is known that novel stimuli activate similar pathways in the brain as emotional stimuli. Specifically the amygdala has been associated with novelty detection and processing (Blackford, Buckholtz, Avery and Zald, 2010). Possibly, via these emotional pathways in the brain, novelty enhanced tilt detection in the present study. A recent functional magnetic resonance imaging (fMRI) study reported that emotional stimuli enhance perceptual processing via amygdala back-projection to the inferior temporal cortex [51], but only when processing *novel* emotional pictures, and not when processing repeated emotional stimuli. The authors concluded that enhanced perceptual processing is triggered by the detection of significance, which decreases when the novelty of a cue diminishes [51]. These results are in line with our findings that novel stimuli can enhance visual perception, and support the notion that the amygdala might play a role in this process. Future studies will have to investigate whether these pathways involving the amygdalae indeed underlie the enhancing effects of novelty on visual perception.
