# Correction: Novelty Enhances Visual Perception

**DOI:** 10.1371/annotation/0e5cfa98-8d8d-4a0f-b489-72886a4ab407

**Published:** 2013-07-30

**Authors:** Judith Schomaker, Martijn Meeter

The city name is incorrect in the authors' affiliation. The correct affiliation is the following:

Department of Cognitive Psychology, VU University, Amsterdam, The Netherlands

